# Will crops with biological nitrification inhibition capacity be favored under future atmospheric CO_2_?

**DOI:** 10.3389/fpls.2023.1245427

**Published:** 2023-08-24

**Authors:** Izargi Vega-Mas, Estefanía Ascencio-Medina, Adrián Bozal-Leorri, Carmen González-Murua, Daniel Marino, María Begoña González-Moro

**Affiliations:** Department of Plant Biology and Ecology, University of the Basque Country (UPV/EHU), Leioa, Spain

**Keywords:** ammonium, biological nitrification inhibitor (BNI), climate change, elevated CO_2_, nitrification, nitrogen fertilization, nitrous oxide (N_2_O), sustainable agriculture

## 1 Introduction

The forthcoming climatic scenario, where elevated atmospheric carbon dioxide (CO_2_) concentrations are expected, will challenge crop performance with a higher demand for nitrogen (N), which will further aggravate N losses from agrosystems that are already polluting air and water systems (Anas et al., 2020; IPCC, 2022), making it imperative to anticipate and develop novel and climate-smart agriculture. Research related to plants showing the ability to produce biological nitrification inhibitors (BNI) as a mitigation strategy is currently in vogue (Saud et al., 2022). Indeed, great progress has been made recently in the characterization of species with this ability, in the production of BNI molecules, and even in the development of new crop lines aimed at incorporating this trait. However, the implications of future environmental conditions on the BNI strategy remain overlooked and need to be addressed. In this study, we aimed to establish the connections between the predicted elevated eCO_2_ conditions and the production and activity of BNI compounds in plants and soil. We hypothesize that enhanced carbon assimilation by plants could improve their BNI capacity, promoting ammonium occurrence in the soil, which would particularly benefit ammonium-adapted crop varieties.

## 2 Nitrogen as centrepiece of plant adaptation to elevated CO_2_

Nitrogen (N) fertilization is required to improve crop yield. However, the inefficiency of agricultural systems, where only 30%–50% of applied N is used by crops, provokes significant losses to the environment in the form of N gas emissions or nitrate (NO_3_ **^−^**) leachates, particularly because of soil microbial nitrification and denitrification (Lassaletta et al., 2014). Nitrification is an aerobic process driven by ammonium-oxidizing bacteria or archaea, where ammonium (NH_4_^+^) is oxidized to NO_3_ **^−^**, which can be further reduced by denitrifying bacteria under anaerobic soil conditions. Both microbial pathways can yield nitrous oxide (N_2_O) as an end-product, which is a powerful GHG (Coskun et al., 2017a). Because N-fertilization is the main source of global anthropogenic N_2_O emissions (IPCC, 2022), great effort has been put into controlling N-cycling processes in agrosystems in recent decades, with the dual aim of maintaining N available for crops for longer periods while reducing its loss to the environment. Therefore, high-production agriculture needs to reconcile the double challenge of mitigating N losses and adapting to progressively changing environmental conditions, such as an elevated atmospheric CO_2_ (eCO_2_) atmosphere, rising temperatures, and water scarcity (FAO, 2018). To this end, climate-resilient crops are required, in a context where more food production will be necessary to maintain the future world population.

The predicted state atmospheric concentration of CO_2_ will reach 600 ppm–1,300 ppm by the end of the century (IPCC, 2022). Elevated atmospheric CO_2_ (eCO_2_) remodels plant physiology, with enhanced photosynthesis and reduced stomatal conductance as the primary effects, leading to improved water use efficiency and potentially boosting plant productivity (Gamage et al., 2018). However, long-term exposure to eCO_2_ often entails photosynthetic acclimation in C3 crops, limiting their growth. Although the physiological basis for acclimation to eCO_2_ is still unclear, one of the most accepted explanations is that increased carbohydrate biosynthesis causes C:N imbalance, leading to N depletion in tissues (Ainsworth and Rogers, 2007). Therefore, acclimation can be overcome by sufficient N supply to ensure proper sink development for excessively formed photoassimilates, thus avoiding RuBisCO inhibition (Ainsworth and Rogers, 2007). In general, using cultivars with enhanced nitrogen use efficiency (NUE) and implementing agricultural practices that ensure soil N availability are advisable to avoid N dilution in plants and maximize crop yields under eCO_2_. Another open debate about the plant response to eCO_2_ is related to the available N source. Several studies have shown similar yield stimulation in response to eCO_2_ regardless of the N form (NH_4_^+^ or NO_3_ **^−^**) assimilated (Vega-Mas et al., 2015; Dier et al., 2018; Andrews et al., 2019). However, some studies have proposed that eCO_2_ inhibits NO_3_ **^−^** assimilation in shoots by diminishing the reducing power of photorespiration (Bloom et al., 2020), while others argue that N limitation at eCO_2_ is a consequence of accelerated growth rather than impaired NO_3_ **^−^** reduction (Andrews et al., 2020; Igarashi et al., 2021). Nonetheless, in view the possible advantage of NH_4_^+^-N sources over NO_3_ **^−^**-N, environmental conditions favoring soil NH_4_^+^ availability to plants would certainly be desirable.

## 3 Biological nitrification inhibition: a promising N-management strategy in a climate change scenario

Increasing N fertilization to address crop N demand in a climate change scenario seems undesirable, as excess soil N could further aggravate the aforementioned water and air pollution (Lassaletta et al., 2014). Therefore, strategies should be developed to promote better utilization of already available N. At present, one of the extensively proven technologies to prolong N retention in soils, while reducing N losses, is the application of synthetic nitrification inhibitors (SNIs) in combination with NH_4_^+^-based fertilizers. The most widely used SNIs are nitrapyrin, dicyandiamide (DCD), and dimethylpyrazol (DMP)-based NIs (Norton and Ouyang, 2019; Huérfano et al., 2022). However, SNIs are not exempt from some disadvantages, including production or management costs that restrict their use, notably in low-income countries, their limited action over time, variable effects on yield, or potential environmental toxicity (Coskun et al., 2017b; Sadhukhan et al., 2022). As a recent alternative, exploitation of the natural capacity of different plants to exudate compounds that suppress microbial nitrification, the so-called biological nitrification inhibitors or BNIs, is a promising strategy (Subbarao and Searchinger, 2021; Lata et al., 2022; Saud et al., 2022). Since the discovery of BNIs in the tropical grass *Brachiaria humidicola* and *Sorghum bicolor* (Subbarao et al., 2007a), the search for plant species displaying this trait has led to the identification of species, including cereals of high agronomical interest such as rice and maize (Tanaka et al., 2010; Sun et al., 2016; Otaka et al., 2022). Wheat cultivars show weak BNI activity but, importantly, the recent development of elite wheat cultivars that harbor a chromosomal region introgressed from *Leymus racemosus*, a wild wheat relative with high BNI activity (Subbarao et al., 2021; Bozal-Leorri et al., 2022), has raised further expectations regarding the potential of crops to directly control nitrification in soils.

How N cycling, and nitrification in particular, will be affected in agrosystems by future climatic conditions, as eCO_2_ is still far from being understood, with variable results shown in the literature (Coskun et al., 2016). In a meta-analysis that included N-fertilized fields, Dijkstra et al. (2012) showed that eCO_2_ led to increased N_2_O emissions due to enhanced nitrification and/or denitrification. High rates of soil nitrification are predicted in the future because nitrifiers use CO_2_ as carbon source for growth and NH_4_^+^ as energy source (Wendeborn, 2020). Indeed, a more abundant nitrifying population was found in response to eCO_2_, alone or in combination with increased temperature (Diao et al., 2020; Waqas et al., 2021). Although the utility of SNIs is unquestionable, their efficiency depends on soil conditions such as water content and temperature (Menéndez et al., 2012; Nair et al., 2021). Bozal-Leorri et al. (2021) recently showed DMP-based SNIs efficiently decreased N_2_O losses regardless CO_2_ level, although further studies are needed to confirm their inhibition efficiency under eCO_2_ in the field and considering different soil types and environmental conditions. Additionally, anticipating how eCO_2_ will affect the plant’s capacity to synthesize and release BNIs, as well as their efficiency in suppressing nitrification, is of great relevance to propose effective strategies to increase NUE by crop plants under future conditions.

## 4 How will eCO_2_ influence plants biological nitrification inhibitory capacity?

**Figure 1:**
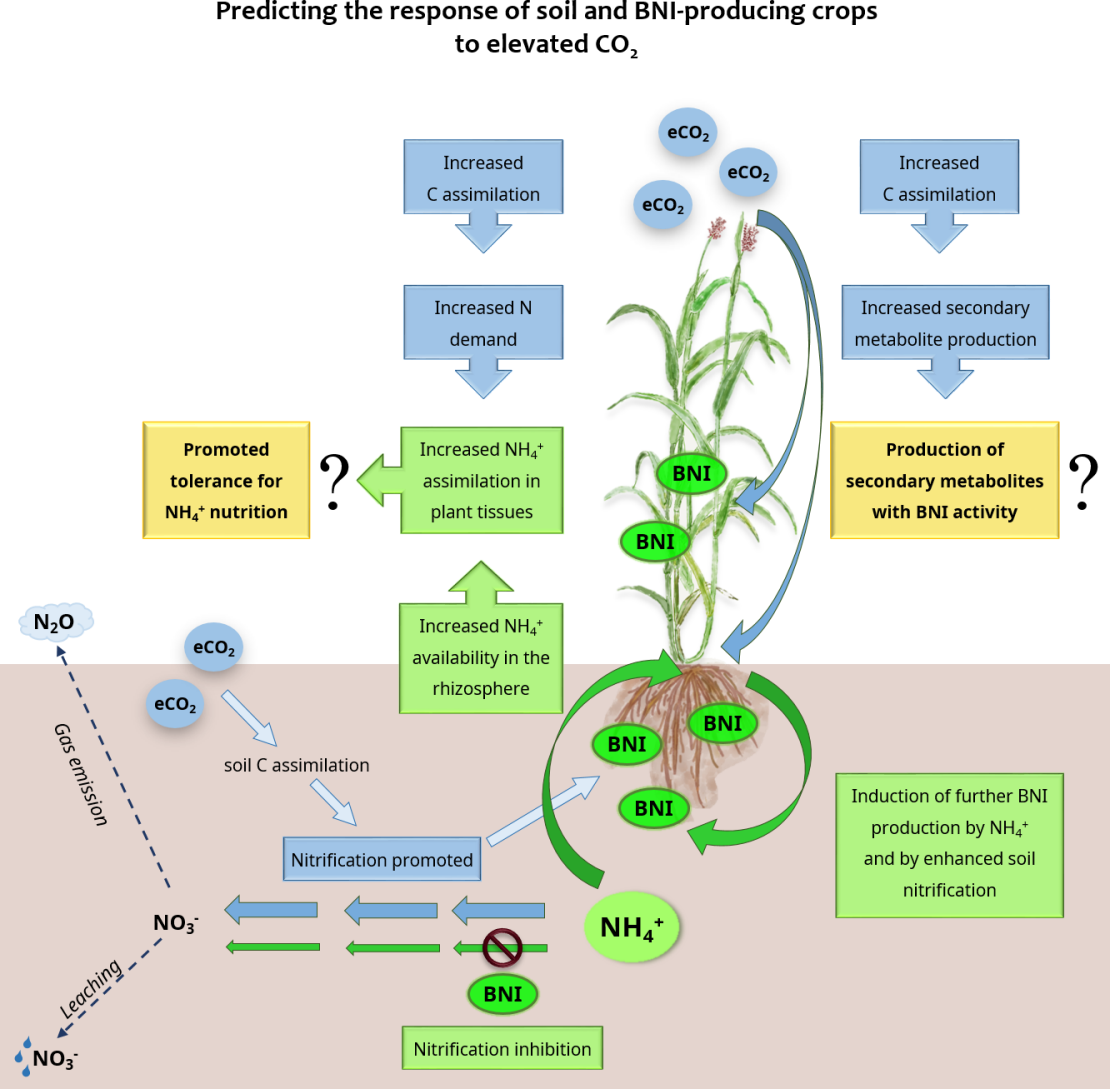
Main view of the predicted effects of elevated CO_2_ levels on soil nitrification and BNI-producing plants. Changes caused by elevated CO_2_ are highlighted in blue, changes due to plant BNI activity are highlighted in green and newly proposed hypotheses are highlighted in yellow.

From an evolutionary point of view, the BNI capacity is considered a plant response to adapt to N-scarce environments (Subbarao et al., 2006; Lata et al., 2022). Conversely, the BNI strategy has also proven to be effective in controlling soil N losses in well N-fertilized systems such as sorghum, rice, and wheat cereal cultures (Subbarao et al., 2021; Wang et al., 2021). Slowing NH_4_^+^oxidation by inhibiting soil nitrification reduces N leakage while promoting NH_4_^+^ stability, thus presumably favoring a more NH_4_^+^-based nutrition. This will surely promote greater yield potential through a more efficient assimilation of co-existent N forms (Subbarao and Searchinger, 2021), which is also crucial to match the enhanced N demands by eCO_2_. Nonetheless, high NH_4_^+^ content in soil may entail a stressful situation for crop performance (Britto and Kronzucker, 2002; González-Moro et al., 2021); hence, crops better adapted to NH_4_^+^ as N source are required. Because plant NH_4_^+^ assimilation is dependent on proper C-skeleton supply, conditions favoring photoassimilate production, such as eCO_2_ or direct carbon provision, have been shown to alleviate the symptoms associated with ammonium stress (Roosta and Schjoerring, 2008; Setién et al., 2013; Vega-Mas et al., 2015). Therefore, the predicted eCO_2_ may be advantageous for improving the performance of BNI-producing plants grown in the presence of enhanced NH_4_^+^ (**Figure 1**).

Plant BNI capacity is dependent on soil conditions, of which rhizospheric pH, aeration, quantity, and form of available N are the main drivers of BNI synthesis and exudation (Wang et al., 2021; Zhang et al., 2022). The present working model indicates that rhizosphere acidification, associated with NH_4_^+^ assimilation and plasma membrane H^+^-ATPase activity, stimulates BNI release (Zhu et al., 2012; Coskun et al., 2017a; Afzal et al., 2020). Hence, more NH_4_^+^-based nutrition would act as a positive feedback regulatory strategy for BNI production and/or release (Subbarao et al., 2007b). Whether plants with higher BNI potential display specific NH_4_^+^-tolerance mechanisms needs to be explored, and results of great interest for the future. Remarkably, the presence of nitrifying bacteria, but not denitrifiers, promotes the secretion of BNI compounds in wheat (O’Sullivan et al., 2016) and rice (Zhang et al., 2019). Thus, although the specific mechanisms responsible for such BNI induction are still unknown, the existence of signaling between BNI-producing roots and nitrifying bacteria has been suggested (Wang et al., 2021). In turn, the predicted promotion of soil nitrification under eCO_2_ conditions (Diao et al., 2020; Waqas et al., 2021) could potentially benefit BNI production.

Elevated CO_2_ promotes not only whole plant and root biomass (Roy and Mathur, 2021), but also root exudate production, which accounts for up to 21% of photosynthetically fixed C (Kollah et al., 2019; Xiong et al., 2019). The BNI compounds identified to date are C-enriched secondary metabolites that belong to a wide range of different metabolic groups, such as quinones, terpenes, and phenolic compounds (Nardi et al., 2020; Chai and Schachtman, 2022). This is the case for BNIs identified as sorgoleone and methyl 3-(4-hydroxyphenyl) propionate (MHPP) from *Sorghum*, or brachialactone from *Brachiaria* (Zakir et al., 2008; Subbarao et al., 2009; Subbarao et al., 2013). Therefore, it would be expected that enhanced root exudation under eCO_2_ to include compounds with BNI activity (**Figure 1**). Overall, secondary metabolites are involved in plant–environment interactions and are produced by plants to ease their adaptation to a changing environment (Zandalinas et al., 2022). Moreover, enhanced net photosynthesis rates under eCO_2_ lead to the rescheduling of secondary metabolism, with enhanced C-enriched metabolite production (Matros et al., 2006; Xu et al., 2019; Roy and Mathur, 2021). Therefore, this reinforces the hypothesis of a possible positive effect of eCO_2_ on the production of BNI-active metabolites. However, root exudation in plants is affected by many factors; water availability is a determinant of exudation response to eCO_2_ (Calvo et al., 2017; Xiong et al., 2019; Chai and Schachtman, 2022). In agreement with the promotion of secondary metabolism under stress conditions, Ghatak et al. (2022) observed that drought stress in pearl millet enhanced the release of root exudates and increased total BNI activity. Deciphering how BNI production is affected by the interaction of factors such as eCO_2_, water availability, or temperature is the next step to further promote this trait for sustainable agriculture.

## 5 Concluding remarks

Many uncertainties still exist in optimizing N management under future climatic conditions. However, to make agriculture more sustainable, it is mandatory to meet crop N demand, while reducing N losses derived from N fertilization. Improving soil N availability through the exploitation of plant BNIs is an outstanding opportunity. In this study, we hypothesize that BNI production would be promoted in a climate change scenario, since eCO_2_ would boost both N assimilation and production of C-rich secondary metabolites. Although there are still many unresolved issues regarding factors that affect plant BNI capacity, BNI crops are promising candidates for future sustainable agrosystem production. In this context, selection of climate-resilient crop varieties adapted to the use of NH_4_^+^ as an N source is essential.

## Author contributions

IV-M and MG-M conceived the manuscript and supervised the whole writing process. All authors listed have made a substantial, direct, and intellectual contribution to the work and approved it for publication.
